# Preoperative Hemoglobin Level Predicts Surgical Site Infections in Trauma Orthopedic Surgery: A Cohort Study

**DOI:** 10.1155/jotm/7737328

**Published:** 2025-02-04

**Authors:** Williams Walana, Fredrick Gyilbagr, Alexis D. B. Buunaaim

**Affiliations:** ^1^Department of Clinical Microbiology, School of Medicine, University for Development Studies, Tamale, Ghana; ^2^Department of Laboratory Service, Tamale Teaching Hospital, Tamale, Ghana; ^3^Department of Surgery, School of Medicine, University for Development Studies, Tamale, Ghana; ^4^Department of Trauma Orthopedics, Tamale Teaching Hospital, Tamale, Ghana

**Keywords:** blood cell parameters, orthopedics, predictors, surgery, surgical site infection, trauma

## Abstract

**Background:** Surgical site infections resulting from trauma orthopedic surgery increase morbidity and mortality rates and generate additional costs for the healthcare system. Preoperative and postoperative blood parameters have been described as risk predictors for surgical site infection in other surgical areas. The purpose of this study was to assess the role of preoperative and postoperative hematological parameters in predicting the risk of surgical site infections in trauma orthopedic surgery.

**Methods:** Data on patients' demographics were collected from their medical records and the operation reports. Preoperative and postoperative blood samples were collected for a complete blood count assay. The blood cell parameters as predictors of surgical site infection after trauma orthopedic surgery were determined by the Mann–Whitney *U* test to assess the differences in the median between the dependent and independent variables. *p* value < 0.05 was considered statistically significant.

**Results:** Out of the 210 patients who were followed postsurgery, 14 (6.7%) developed surgical site infection following trauma orthopedic surgery. The mean age of the study participants was 33.08 ± 19.23 (Mean ± SD), with a range of 86 to 0.67 years old. Low preoperative hemoglobin level was identified as a predictor of surgical site infection following trauma orthopedic surgery (*p*=0.019). None of the postoperative blood parameters measured was significantly associated with surgical site infections after trauma orthopedic surgery in Northern Ghana.

**Conclusion:** In conclusion, our study demonstrates that preoperative hemoglobin level is a useful hematological parameter for predicting surgical site infection following trauma orthopedic surgery. These inexpensive and common hematological parameters could assist in guiding preventive efforts to reduce surgical site infections and improve outcomes for vulnerable patients undergoing trauma orthopedic surgery. Assessing preoperative hemoglobin levels is crucial in identifying patients at increased risk of developing surgical site infections. Preoperative optimization, including incorporating hemoglobin levels into predictive risk models can help to assess these at-risk persons better. Educate patients on the need to optimize their hemoglobin levels before surgery and discuss potential interventions, including iron supplementation or transfusion.

**Summary**

• Low preoperative hemoglobin level was identified as a predictor of surgical site infections following trauma orthopedic surgery in Northern Ghana.
• None of the postoperative blood parameters measured was significantly associated with SSIs after trauma and orthopedic surgery in Northern Ghana.

## 1. Introduction

Surgical site infection (SSI) is an adverse complication of orthopedic surgery and can increase the risk of readmission [1, 2]. Poor prognosis, a reduced quality of life, and the potential for reoperation are further consequences of SSI [3, 4]. The Centers for Disease Control and Prevention (CDC) classifies SSIs into three groups [5]. These include Superficial SSIs that are limited to the skin and subcutaneous tissue, Deep Incisional SSIs involving the fascia and muscle layers, and Organ or space SSIs connected to the human organs and body spaces. With incidence rates ranging from 0.4 to 30.9 per 100 patients undergoing surgery and a pooled incidence rate of 11.8 per 100 patients undergoing surgery, SSI continues to be one of the most common healthcare-acquired infections in low- and middle-income countries (LMICs) [6]. These rates are noticeably higher than those seen in developed nations.

Several strategies have been implemented to prevent SSIs, including enhancing general orthopedic surgical procedures, raising anesthesiology standards, and lowering SSI-related risk factors [7]. Despite these efforts to prevent SSI, the incidence of SSI persists and varies from 0.7% to 11.9%, depending on the procedure's complexity and type of surgery, as described by a previous study [8]. Thus, besides an initial diagnosis by a surgeon or assessment with imaging equipment, which can effectively determine whether SSI occurs or not. The prognosis and monitoring of SSI can also benefit greatly from other indicators, such as pertinent clinical laboratory markers [9, 10].

Since the routine blood count is the most basic and rapid detection method that can immediately provide insight into the patient's condition, we hypothesized that blood count parameters may be utilized for predicting SSI after orthopedic surgery. One of the easiest, most reliable, and least expensive diagnostics for patients waiting on orthopedic surgery is the complete blood count (CBC). Hemoglobin (Hb), eosinophil count, platelet count, and other preoperative and postoperative CBC indicators have been shown to correlate with patient survival in addition to directing the therapeutic management of surgical candidates [11, 12].

SSIs can generally be prevented by addressing the underlying risk factors. By identifying these risk factors for SSIs, postoperative outcomes and quality of life can be improved, and the re-admission rate can be decreased [13–15]. However, there is still limited data in this setting regarding the use of preoperative and postoperative CBC parameters as predictors of postoperative infections following trauma orthopedic surgery. Considering these, this study was conducted to assess the role of preoperative and postoperative blood parameters in predicting the risk of SSI.

## 2. Methodology

### 2.1. Ethical Consideration

This study received approval from the institutional review board of the University for Development Studies (UDS/IRB/127/23). Site permission was granted by the Tamale Teaching Hospital (TTH) and the Department of Surgery (TTH/R&B/SR/283). Patients' participation was strictly voluntary, and informed consent was granted by patients.

## 3. Study Area

This study was conducted at the TTH, located in Tamale in the Northern Region of Ghana, and serves as a referral hospital for the sector (Northern, Savana, North-East, Upper East, and Upper West). The hospital also treats patients who have been referred from Togo, Burkina Faso, and Mali (TTH Administration Directorate, 2015). The choice of this medical center was made in response to the considerable number of orthopedic patients it receives, the diverse patient population, and the predicted growth in cases brought on by the expansion of motorized transportation in this region of Ghana.

The TTH has a bed capacity of 800 and offers general medicine, surgery, obstetrics and gynecology, pediatrics, orthopedics, ophthalmology, dermatology, psychiatry, and other services. The hospital contains specialized departments and sections that are staffed by skilled medical staff and administrative personnel who are dedicated to giving patients high-quality care. In addition to offering clinical services, the TTH is actively engaged in research, which advances knowledge and promotes healthcare.

## 4. Study Design, Inclusion, and Exclusion Criteria

A prospective study was conducted at the TTH from September 2023 to May 2024. Selected participants comprised patients who had undergone surgery at the trauma and orthopedics surgical ward. We excluded patients admitted to the orthopedic surgical department but did not undergo any surgery and those who died after surgery. In addition, patients who had their wound infected before surgery (diagnosed by the surgeon and confirmed by culture-positive results) were excluded from this study. Also, patients with incomplete data of medical records by opting out within 6 months postsurgery were excluded.

## 5. Data Collection

The comprehensive data collection instrument was designed to gather demographic characteristics of the patients and the blood cell parameters both pre- and postsurgery. The questionnaires were based on the objectives of the study; hence they were designed into themes according to the objectives. The demographic characteristics considered include age, gender, marital status, educational level, occupation, religious background, and residential area. The blood cell parameters considered include for both pre- and postsurgery total white blood cell count (WBC) count, platelets count, lymphocyte differential count, neutrophils differential count, Hb levels, and neutrophil/lymphocyte ratio (NLR).

## 6. Samples Collection

For this study, blood samples were collected from study participants before surgery and postday 3. After collection, all the samples are carefully labeled with patient details and transported within 30 min to the hematology laboratory at the TTH for CBC or full blood count (CBC/FBC) analysis. For those who were suspected to have developed an infection, following the CDC criteria for diagnosis of SSI, the suspected patients had their wounds cleaned with normal saline before the samples were taken. Wound swabs, fluid, or aspirate were aseptically collected from patients using a sterile cotton-tipped applicator or a syringe. The samples are labeled with patient details and transported in Stuart transport media within 1 h to the microbiology laboratory at the TTH for bacteria culture and sensitivity, and Ziehl Neelsen staining (for *Mycobacterium tuberculosis*) testing to be done.

## 7. Definition of Surgical Site Classifications

The SSI was classified according to the CDC. The center classifies SSIs into three groups [5]. These include.

### 7.1. Superficial Incisional SSI

Infection occurs within 30 days after the operation and involves only the skin or subcutaneous tissue of the incision.

### 7.2. Deep Incisional SSI

Infection occurs within 30 days after the operation if no implant is left in place or within 1 year if an implant is in place and the infection appears to be related to the operation and involves deep soft tissues (e.g., fascial and muscle layers) of the incision.

### 7.3. Organ/Space SSI

Infection occurs within 30 days after the operation if no implant is left in place or within 1 year if an implant is in place and the infection appears to be related to the operation; and the infection involves any part of the anatomy (e.g., organs or spaces), other than the incision, that was opened or manipulated during the operative procedure.

## 8. Statistical Analysis

This data was analyzed using SPSS version 27. Frequencies, percentages, and cross-tabulations were used to summarize the demographic variables. We employed normality testing to determine whether the data were a normally distributed population, but the output turned out to be skewed. We utilized the Mann–Whitney *U* test which is a nonparametric statistical tool to assess the differences in the median between the dependent and independent variables. *p* value < 0.05 was considered statistically significant in determining the predictors of SSI following trauma orthopedic surgery. The reference ranges for Hb concentrations were as follows: (1) Male: 14–18 g/dL (SI units), (2) Female: 12–16 g/dL (SI units), (3) Pregnant female: > 11 g/dL [16].

## 9. Results

### 9.1. Sociodemographic Characteristics and Incidence of SSIs Among the Study Participants

A total of 14 patients developed SSI following TOS 6.7% (14/210). None of the sociodemographic characteristics considered in this study showed a significant association with SSI following TOS. The mean age of the study participants was 33.08 ± 19.23 (Mean ± SD), with a range of 86 years and 0.67 years. However, the predominant age groups were between 21–30 and 41–50 years (18.6% and 19.0% respectively). Regarding gender, males were predominant (68.6%), and many of the study participants were married (55.7%). Education-wise, about a quarter had tertiary level education, while 19.0%, 12.9%, and 11.4% had, respectively, primary, JHS, and SHS levels of education. Comparatively, the study participants resided in the rural area (42.4%), 36.7% in the urban and 21.0% peri-urban area settlers, Table 1.

### 9.2. Preoperative and Postoperative Blood Parameters in Predicting SSI Following TOS

Out of the 210 patients recruited for this study, 157 had their blood samples taken at different times for preoperative and postoperative (post-of-day 3) CBC analysis. The preoperative and postoperative blood parameters considered were total WBC, Hb level, platelets count, neutrophils differential count, lymphocytes differential count, and NLR. However, preoperative Hb was strongly linked to SSI and as such identified as a predictor of SSI following trauma orthopedic surgery (*p*=0.019). On the contrary, none of the postoperative parameters were linked to SSI following trauma orthopedic surgery (*p* values > 0.05) as shown in Table 2.

Mann–Whitney *U* test was used to compare the differences in medians between the two variables and the output are displayed: Median (Interquartile Range) and *p* values of < 0.05 was considered statistically significant.

### 9.3. Pre-Existing Conditions/Factors of SSI After TOS

The pre-existing conditions and other lifestyle factors considered were diabetes mellitus, hypertension, smoking, alcohol use, asthmatic, congestive cardiac failure, cancer patient, and the American Society of Anesthesiologists (ASA) score. The results revealed that none of the factors was significantly associated with SSI following TOS (*p* value > 0.05) as shown in Table 3.

## 10. Discussion

Among the most prevalent nosocomial infections in orthopedic surgical patients is SSI. It is a dangerous surgical consequence that happens after about 2% of surgeries and makes up 20% of infections linked to healthcare [17]. SSI greatly increases the length of a patient's postoperative hospital stay while also raising the cost of care, inflicting a heavy financial burden on the patient [18]. The current study showed a higher proportion of males than females (ratio 2.2:1). This can be explained by the fact that males are more involved in outside activities and field occupations putting them at higher risk of accidents with fractures and other related injuries. This predominance of males in operated patients has been previously illustrated in other studies [19–21].

For comorbidities and other related conditions that predispose patients to SSI following TOS, the results revealed that none of the factors was significantly associated with SSI. This finding is in contrast to a report that identified poorly controlled diabetes mellitus, chronic alcoholism, and ASA score as independent risk factors of SSI following TOS [22].

This study reported no statistically significant between preoperative leukocytes and SSI. This is in line with a study conducted in Switzerland who reported preoperative leukocytes as not significantly associated with SSI [23]. In contrast, other studies have found a significant association between preoperative leukocytes and postoperative infections [24, 25]. This leukocytosis may be caused by a pre-existing infection, posing a higher risk of SSI after surgery. It could also be associated with malnutrition or other risk factors that render the individual at a higher risk of SSI. In this study, the preoperative NLR was not significantly associated with SSI. This is in contrast with a study conducted in Turkey which identified preoperative NLR as significantly associated with SSI [26]. The differences could be due to variations in preoperative care, including the use of prophylactic antibiotics, nutritional support, hygiene practices, timing of blood sample collection, surgical techniques, and definitions of SSI, which could introduce variability [27].

In this study, postoperative neutrophils, postoperative lymphocytes, and postoperative NLR show no significant association with SSI. This is in contrast with a study conducted in Japan [28] where these parameters showed a significant association with SSI. Another study also showed that NLR postoperatively could significantly discriminate between SSI and non-SSI groups after orthopedic surgery [29]. Thus, using NLR would offer some diagnostic utility for the early prediction of SSI after orthopedic surgery. However, the results of our study may also indicate that NLR provides no advantage for the early prediction of SSI after orthopedic surgery. A few factors, such as the participants' selection, the sampling time, surgical techniques, implant used blood loss, surgical complexity and duration of surgery could contribute to the variation.

Platelets are small, non-nucleated cellular debris that circulates in the blood, and their roles in thrombosis and hemostasis are well established. Inherited or acquired platelet count defects or function defects may be associated with bleeding complications [30]. In recent years, many studies have highlighted that platelets are associated with certain infectious and inflammatory diseases [31, 32]. A previous study proposed that the platelets count distribution can be used as an important additional detection method for the diagnosis of SSI after traumatic orthopedic surgery, thereby reducing the cost and time loss [33]. However, the present study identified no significant association for both preoperative platelets and postoperative platelets count of SSI following trauma orthopedic surgery. The differences could be due to a variety of factors, including differences in study design, patient characteristics, infection diagnostic criteria, and statistical methodologies. The relationship between platelet count and SSI is complex and may involve multiple factors beyond just the platelet count, such as platelet function, inflammatory pathways, and patient-specific variables [34].

The present study identified preoperative Hb as a predictor of SSI following trauma orthopedic surgery (*p* value < 0.05). This is in line with previous studies which reported preoperative Hb as significantly associated with SSI following trauma orthopedic surgery by [35, 36]. This could probably be because low Hb levels can reduce oxygen tension and affect collagen formation. This impairs macrophage function and stops the healing process from progressing, increasing the risk of wound infection. Since it produces a less stable scar and favors dehiscence and infection [37]. However, a previous study conducted also revealed that preoperative Hb showed no significant association with SSI [38]. The present study identified no significant association between postoperative Hb and SSI following trauma orthopedic surgery. In contrast, a previous study reported postoperative Hb as a predictor of SSI [39].

## 11. Conclusion

This study established a 6.7% (14/210) incidence of SSI following TOS in northern Ghana. Additionally, our findings suggest that preoperative Hb is a helpful marker for predicting SSI following trauma orthopedic surgery. Hematological parameters are necessary indicators for perioperative monitoring. We can pay more attention to preoperative Hb when monitoring preoperative blood parameters in relation to SSI. Measuring Hb levels before surgery is essential for identifying patients who are at higher risk of developing SSIs. Hb levels can be used in predictive risk models as part of preoperative optimization to help better determine an individual's risk of developing SSI. Inform patients about the need to optimize their Hb levels before surgery and provide possible remedies, such as iron transfusions or supplements. Expanding this study may have implications for identifying specific predictors of SSI after trauma orthopedic surgery.

### 11.1. Limitation of the Study

This cohort study is relatively small and may affect the generalization of our findings. Further studies are essential in larger cohorts to expatiate and validate these findings. Future studies should be expanded to capture the differences in preoperative and postoperative Hb levels, and expand the scope of the blood parameters among these groups of patients.

## Figures and Tables

**Table 1:** Sociodemographic characteristics of study participants.

Variables	Frequency (*n* = 210)	Percentage (%)
Age categories (years)		
≤ 10	27	12.9
11–20	35	16.7
21–30	39	18.6
31–40	33	15.7
40–50	40	19.0
> 50	36	17.1
Gender		
Male	144	68.6
Female	66	31.4
Marital status		
Married	117	55.7
Single	81	38.6
Divorced	4	1.9
Cohabitation	8	3.8
Educational level		
No education	68	32.4
Primary	40	19.0
Junior high school	27	12.9
Senior high school	24	11.4
Tertiary/above	51	24.3
Occupation		
Government worker	29	13.8
Private worker	29	13.8
Self-employment	37	17.6
Student	50	23.8
Unemployment	65	31.0
Religious background		
Christians	48	22.9
Muslims	150	71.4
Traditionalist	12	5.7
Residential area		
Urban	77	36.7
Peri-urban	44	20.9
Rural	89	42.4

**Table 2:** Preoperative and postoperative blood parameters in predicting SSI following TOS.

Variables	SSI (*N* = 14)	No SSI (*N* = 143)	*p* value
Preoperative variables			
Preoperative Hb	10.35 (8.78–11.28)	11.30 (9.60–12.80)	**0.019**
Preoperative WBC	9.86 (5.48–11.32)	8.70 (6.14–11.42)	0.397
Preoperative platelets	224 (173.75–268.75)	222 (165–299)	0.925
Preoperative lymphocytes	1.72 (1.42–2.95)	2.47 (1.75–4.82)	0.826
Preoperative neutrophils	6.23 (3.09–9.10)	4.02 (2.54–6.86)	0.510
Preoperative NLR	2.79 (1.73–5.90)	1.53 (0.67–3.30)	0.363
Postoperative variables			
Postoperative Hb	9.45 (8.35–10.65)	9.60 (8.40–11.02)	0.177
Postoperative WBC	8.51 (7.20–12.36)	9.34 (7.05–11.02)	0.730
Postoperative platelets	276.50 (196–380.25)	307 (207–405)	0.683
Postoperative lymphocytes	2.53 (1.73–3.26)	3.21 (2.30–4.60)	0.397
Postoperative neutrophils	5.68 (4.71–7.69)	4.95 (3.24–7.52)	0.778
Postoperative NLR	2.29 (1.66–5.12)	1.66 (0.81–2.99)	0.638

*Note:* Bold values mean statistically significant.

Abbreviations: Hb = hemoglobin, L = lymphocyte, N = neutrophils, NLR = neutrophils–lymphocytes ratio, and WBC = white blood cells.

**Table 3:** Pre-existing conditions/factors of SSI after TOS.

Variables	Total	Surgical site	Infection	*p* value
		Yes (*n* = 14)	No (*n* = 196)	
Diabetes mellitus				0.641
Yes	3 (1.4)	0 (0.0)	3 (1.5)	
No	207 (98.6)	14 (100.0)	193 (98.5)	
Hypertension				0.646
Yes	22 (10.5)	2 (14.3)	20 (10.2)	
No	188 (89.5)	12 (85.7)	176 (89.8)	
Smoking				0.704
Yes	2 (1.0)	0 (0.0)	2 (1.0)	
No	208 (99.0)	14 (100.0)	194 (99.0)	
Alcohol used				0.641
Yes	3 (1.4)	0 (0.0)	3 (1.5)	
No	207 (98.6)	14 (100.0)	193 (98.5)	
Asthmatic				0.704
Yes	2 (1.0)	0 (0.0)	2 (1.0)	
No	208 (99.0)	14 (100.0)	194 (99.0)	
Congestive cardiac failure				0.789
Yes	1 (0.5)	0 (0.0)	1 (0.5)	
No	209 (94.5)	14 (100.0)	195 (99.5)	
Cancer patient				0.641
Yes	3 (1.4)	0 (0.0)	3 (1.5)	
No	207 (98.6)	14 (100.0)	193 (98.5)	
ASA score				0.958
ASA 1	179 (85.2)	12 (85.7)	167 (85.2)	
ASA 2	27 (12.9)	2 (14.3)	25 (12.8)	
ASA 3	3 (1.4)	0 (0.0)	3 (1.5)	
ASA 4	1 (0.5)	0 (0.0)	1 (0.5)	

*Note:* ASA: American Society of Anesthesiologists physical status classification system.

## Data Availability

The data that support the findings of this study are available from the corresponding author upon reasonable request.
